# Grating-based phase-contrast and dark-field computed tomography: a single-shot method

**DOI:** 10.1038/s41598-017-06729-4

**Published:** 2017-08-07

**Authors:** Maximilian von Teuffenbach, Thomas Koehler, Andreas Fehringer, Manuel Viermetz, Bernhard Brendel, Julia Herzen, Roland Proksa, Ernst J. Rummeny, Franz Pfeiffer, Peter B. Noël

**Affiliations:** 1 Chair of Biomedical Physics, Department of Physics and Munich School of Bioengineering, Technical University of Munich, 85748 Garching, Germany; 2 Philips GmbH Innovative Technologies, Research Laboratories, 22335 Hamburg, Germany; 3 Institute for Advanced Study, Technical University of Munich, 85748 Garching, Germany; 4 Department of Diagnostic and Interventional Radiology, Klinikum rechts der Isar, Technical University of Munich, 81675 Munich, Germany

## Abstract

Grating-based X-ray interferometry offers vast potential for imaging materials and tissues that are not easily visualised using conventional X-ray imaging. Tomographic reconstruction based on X-ray interferometric data provides not only access to the attenuation coefficient of an object, but also the refractive index and information about ultra-small-angle scattering. This improved functionality comes at the cost of longer measurement times because existing projection-based signal extraction algorithms require not only a single measurement per projection angle but several with precise grating movements in between. This obstacle hinders the adaptation of grating-based interferometry into a continuously rotating gantry. Several solutions to this problem have been proposed but all suffer from major drawbacks. We present results using an iterative reconstruction algorithm working directly on the interferograms. The suggested direct approach enables improved image quality, since interpolations and unnecessary assumptions about the object are circumvented. Our results demonstrate that it is possible to successfully reconstruct the linear attenuation coefficient, the refractive index and the linear diffusion coefficient, which is a measure related to ultra-small-angle scattering, using a single measurement per projection angle and without any grating movements. This is a milestone for future clinical implementation of grating-based phase-contrast and dark-field contrast X-ray computed tomography.

## Introduction

Computed Tomography (CT), a method which combines a series of X-ray images taken from different angles and uses computer processing to create cross-sectional images, is the backbone of current diagnostic medical imaging. At the same time, CT has several limitations, such as low soft-tissue contrast, as is necessary for the detection of, for example, oncological lesions, and a limited sensitivity to structural changes in the tissue, necessary for the early detection of, for example, pathological changes of the lung parenchyma due to chronic obstructive pulmonary disease (COPD).

On the one hand X-ray phase-contrast imaging (PCI) offers the potential for increased soft-tissue contrast due to a more significant difference between different types of soft tissue in the refractive index than in the attenuation coefficient^1^. On the other hand, X-ray dark-field imaging (DFI) offers a tool which can assess structural changes within a sample as the contrast in DFI is created by ultra-small-angle scattering at microstructures of a much smaller scale than the spatial resolution of the imaging system^2^. For homogeneous objects that show no or negligible ultra-small-angle scattering, the dark-field signal is close to zero, whereas strongly scattering samples yield a significant signal. This way, the dark-field images reveal sub-resolution structural information on the nanometer and micrometer length scale that is inaccessible using conventional attenuation X-ray imaging^2–4^. In previous studies, it has been demonstrated on mice that dark-field imaging can improve the detection and quantification of several lung diseases such as emphysema^5^ or fibrosis^6^. The majority of these studies have been conducted in projection mode, but recently the technology has also been applied to assess lung disease in living mice using dark-field CT (DFCT)^7^.

For the successful translation of both PCI and DFI into a clinical full body CT scanner, several essential challenges remain. For example, current clinical standards for radiation exposure and acquisition speed need to be met by future systems. One of the most promising concepts for clinically accessing the signals is grating-based X-ray imaging and the CT equivalent, grating-based computed tomography (GBCT)^8^. This technique is based on the introduction of a three-grating interferometer into the beam path, so that the the signal measured on the X-ray detector is sensitive to X-ray attenuation, refraction and ultra-small-angle scattering. A single recording of such an interference pattern is called an interferogram. For conventional GBCT, at least three interferograms per projection angle must be measured to be able to extract attenuation, dark-field, and differential phase-contrast projection images. For each of these measurements it is essential to position the gratings with an accuracy well below the grating pitch (usually in the range of a few microns)^9^. This procedure is called phase-stepping and makes the translation of GBCT into a continuously rotating gantry—a clinical must—impossible.

Several solutions have been proposed to overcome this challenge, but those solutions come with shortcomings in terms of a clinical translation. For example, the reverse projection method^10^ combines two interferograms taken exactly 180 degrees apart. This method poses very strict requirements on the stability of the grating alignment and gives only access to attenuation and differential phase-contrast projection images. Additionally, it is unclear if this technique can be adapted to systems with significant cone-beam geometry and helical acquisition protocols. The Moiré analysis method^11, 12^ is a true single-shot technique that uses Fourier analysis to detect subtle changes caused by the measured object on a high-frequency Moiré fringe pattern created from slight detuning of the interferometer. This approach does not require grating movements but suffers from a significant loss in resolution in one dimension of the detector. A different approach, which is a direct successor of traditional phase-stepping, is the sliding window method^13^. This technique requires only a single interferogram per projection angle, but changes the grating position at each angular position. To mimic a classical phase-stepping scan, the missing interferograms are simply interpolated from adjacent projection angles. This works well for scans with high angular sampling but if the number of projection angles is reduced, the interpolation step causes blurring and artifacts. In addition to techniques that require only a single interferogram per projection angle, a solution that overcomes the need for precise mechanical grating movements (but still requires multiple measurements per angular position) has been presented: Electromagnetic phase-stepping (EPS)^14^ employs electrical beam steering to move the object’s projection slightly across the detector while a deliberately created Moiré fringe pattern is fixed on the detector. Several of these shifted images can be combined to form a pseudo phase-stepping.

All these solutions, including traditional phase-stepping, have in common that they calculate sets of attenuation, differential phase-contrast and dark-field projections for each projection angle from the interferograms. These three sets are then separately reconstructed to generate three independent volumes of the underlying object properties: the attenuation projections contain information about the spatial distribution of the linear attenuation coefficient, the differential phase-contrast projections are connected to the refractive index decrement, and the dark-field projections allow reconstruction of the linear diffusion coefficient^3^.

Recently a novel approach for the reconstruction of data acquired at a grating interferometer has been proposed and demonstrated on simulations and a real scan using phase-stepping acquisition patterns^15, 16^. In this approach the whole interferometric image formation process is incorporated into the forward model of a statistical iterative reconstruction (SIR) algorithm. This allows for a simultaneous reconstruction of all three object properties based directly on the measured interferograms (IBSIR). In this work we present two methods that combine the IBSIR reconstruction algorithm with a sliding window acquisition pattern (SW-IBSIR) and with a single-shot version of the electromagnetic phase-stepping (SSEPS-IBSIR). The successful application of these techniques demonstrates that using the right reconstruction approach and a suitable acquisition pattern allows us to eliminate the most critical challenges in GBCT. These challenges include the need for multiple measurements per projection angle, and precise grating positioning during the scan, without a loss of image quality. In the near future, a clinical translation of GBCT to a continuously rotating gantry may become feasible.

## Results

### Reconstruction algorithm and acquisition patterns

The algorithm we used to simultaneously reconstruct the distribution of the linear attenuation coefficient *μ*, the refractive index decrement *δ*, and the linear diffusion coefficient *ε* of an object, is outlined in the following. It is based on iterative minimization of a cost function depending on these three quantities. It consists of a data term *L*, measuring the likelihood for the reconstructed image to belong to the measurement^15^, and a regularization term *R* that favors a smooth solution^16^

1 $$C(\mu ,\delta ,\varepsilon )=L(\mu ,\delta ,\varepsilon )+R(\mu ,\delta ,\varepsilon \mathrm{).}$$

The values $\hat{\mu }$, $\hat{\delta }$, and $\hat{\varepsilon }$, that minimize the cost function for a given set of measurements, are the best estimates for the distribution of *μ*, *δ*, and *ε* according to the maximum a posteriori (MAP) principle.

For energy integrating X-ray detectors and for photon counting detectors at sufficient flux we can assume that the measured intensities *I*_*i*_ are Gaussian distributed around their expected values ${\bar{I}}_{i}$ with variance ${\sigma }_{i}^{2}$. For this case, the joint negative log-likelihood (without any prefactors) to make the measurement *I* can be written as

2 $$L(I|\bar{I})=\sum _{i}\frac{1}{{\sigma }_{i}^{2}}{({I}_{i}-{\bar{I}}_{i})}^{2}\mathrm{.}$$

The index *i* denotes a specific detector element at a specific angular and grating position.

The forward model for ${\bar{I}}_{i}$ is dependent on the distribution of *μ*, *δ*, and *ε* and can be written as

3 $${\bar{I}}_{i}={I}_{i}^{0}{T}_{i}[1+{V}_{i}^{0}{D}_{i}{\cos ({\rm{\Phi }}}_{i}^{0}-{{\rm{\Phi }}}_{i})],$$

where *I* ^0^, *V* ^0^, and Φ^0^ are mean intensity, visibility, and phase position of a blank scan without the sample present. The transmission *T*, dark field signal *D*, and differential phase Φ are related to the object by

4 $${T}_{i}=\exp [-{\int }_{{l}_{i}}\mu (x)dx],$$

5 $${D}_{i}=\exp [-{\int }_{{l}_{i}}{c}_{D}\varepsilon (x)dx],$$

6 $${{\rm{\Phi }}}_{i}={c}_{\varphi }{\partial }_{y}{\int }_{{l}_{i}}\delta (x)dx,$$

where ${\int }_{{l}_{i}}dx$ is the line integral from pixel *i* to the x-ray source and ∂_*y*_ is the partial derivative perpendicular to the grating direction. To extract quantitatively comparable results from the reconstructions, specific setup geometry constants *c*_*D*_ and *c*_*ϕ*_ must be considered^3, 17^.

The regularizer term *R* consists of three single regularizers *R*_*μ*_, *R*_*δ*_, and *R*_*ε*_ with corresponding regularization strength parameters *β*_*μ*_, *β*_*δ*_, and *β*_*ε*_, which enforce a smooth solution in each of the reconstructed volumes.

7 $$R=\sum _{\theta \in \{\mu ,\delta ,\varepsilon \}}{\beta }_{\theta }{R}_{\theta }\mathrm{.}$$

Further information about the reconstruction algorithm can be found in the methods section.

Three different acquisition patterns were used to record the scans that were reconstructed using the IBSIR method: Equidistant phase stepping (PS), sliding window phase-stepping (SW), and a version of the electromagnetic phase-stepping acquisition where only a single image is acquired at each angular position (SSEPS). For the PS acquisition, *N*_*PS*_ measurements are taken at each angular scan position and one of the interferometer gratings is shifted by 1/*N*_*PS*_ of its grating period after each measurement. For the SW acquisition only a single measurement is taken at each angular scan position but the grating position of one of the gratings is shifted by a fraction of its period. The SSEPS scan pattern takes only a single measurement at each angular position but the position of the focal spot of the X-ray tube is shifted after each measurement. This causes the projection of the measured object to move across the detector while the Moiré fringe pattern stays fixed on the detector.

### Single-shot GBCT simulation

**Figure 1:**
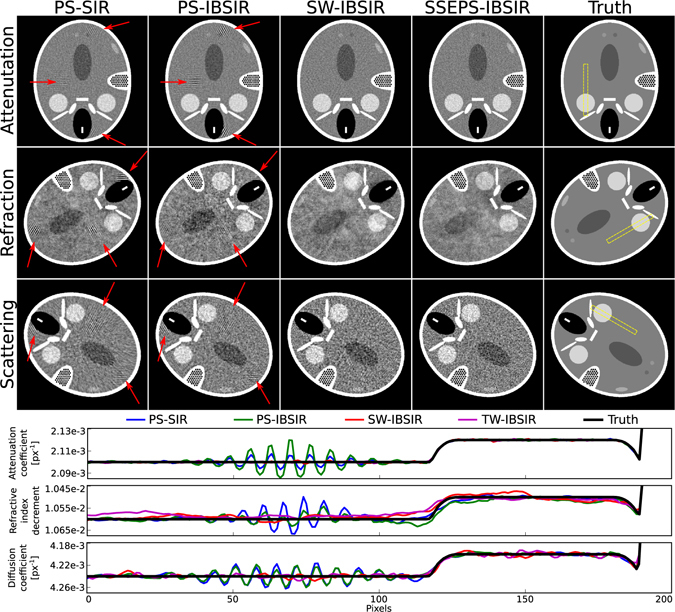
Comparison of different reconstruction methods for several acquisition patterns. The rows from top to bottom show reconstructions of a phantom’s attenuation, refractive index decrement and linear diffusion coefficient. (PS-SIR) shows reconstructions of a phase-stepping scan composed of 101 angular positions with 3 phase-steps each. (PS-IBSIR) shows reconstructions of the same scan using a direct interferogram-based reconstruction algorithm. Both reconstructions show distinct artifacts, marked by the arrows, caused by angular undersampling. (SW-IBSIR) uses the IBSIR algorithm to reconstruct a sliding window acquisition pattern composed of 303 angular positions using only a single, but changing, grating position. (SSEPS-IBSIR) is the reconstruction of a single-shot electromagnetic phase-stepping acquisition pattern using a single fixed grating position for 303 angular positions. Both methods strongly reduce the undersampling artifacts. Below the images line plots along the yellow box for all channels are given.

The performance of the proposed method is first demonstrated on numerical simulations. The simulated phantom exhibits X-ray attenuation, refraction, and ultra-small-angle scattering. To create this phantom the FORBILD head phantom^18^ was extended to three sub-phantoms with a well-defined linear attenuation coefficient *μ*, refractive index decrement *δ*, and linear diffusion coefficient *ε* for each material component. The phantom contains high-contrast elements, which are small in size and whose reconstruction is prone to undersampling artifacts, and large low-contrast elements that are challenging to reconstruct correctly at the same time. Rotating the sub-phantoms with respect to each other (see ground truth in Fig. 1) allowed us to better attribute which property of the phantom was causing image artifacts. This destroys any anthropomorphology of the phantom but, for example, if all elements were perfectly co-aligned, artifacts from photon starvation caused by high attenuation would be visible in all three volumes, but would not be unambiguously attributable to the attenuation property because at the same location the phantom also exhibits high ultra-small-angle scattering and refraction. All simulations were performed on a 500 × 500 × 5 voxel volume.

Scans with three different acquisition patterns (PS, SW, and SSEPS), each using the same total exposure time and number of detector readouts, were simulated. The PS scan used three different grating positions at each of 101 angular positions equally distributed over 360 degrees. The SW scan took measurements at each of 303 angular positions equally distributed over 360 degrees and after each measurement the grating position was shifted by 1/3 of the grating period. For the SSEPS scan, 303 measurements equally distributed over 360 degrees were also simulated, and the focal spot position of the X-ray tube was shifted by one pixel after each measurement. More details on the simulations can be found in the methods section.

The simulations were reconstructed using several methods: The PS scan was processed into attenuation, differential phase-contrast and dark-field projection images. Then the different sets of projections were reconstructed using filtered backprojection (FBP) and statistical iterative reconstruction (SIR)^17, 19–22^. Additionally, this scan was directly (without previous signal extraction) reconstructed using the IBSIR method. The PS-SIR and PS-IBSIR reconstructions are shown in Fig. 1. The PS-FBP reconstruction produced vastly inferior results with strong undersampling artifacts (the image quality metrics can be found in Table 1). PS-SIR and PS-IBSIR reveal very comparable reconstruction results. Both show slight but clearly visible undersampling artifacts originating at the high-frequency high-contrast areas of the phantom in all modalities. Similar image quality was expected as both scans are based on the same underlying information. In this specific case, adequate photon statistics and sufficient sampling of the stepping curve enabled the signal extraction step to correctly extract the underlying signals and to correctly propagate the statistical weights for the SIR from the raw measurements.

**Table 1:** Normalized root-mean-squared error (NRMSE), normalized mean absolute error (NMAE), and mean structural similarity (MSSIM) were separately calculated for the linear attenuation coefficient *μ*, refractive index decrement *δ*, and linear diffusion coefficient *ε*.

	NRMSE in %			NMAE in %			MSSIM		
	*μ*	*δ*	*ε*	*μ*	*δ*	*ε*	*μ*	*δ*	*ε*
PS-FBP	26.0	24.9	26.6	19.1	18.0	19.9	0.556	0.557	0.558
PS-SIR	0.411	0.742	3.98	0.251	0.393	0.793	0.731	0.687	0.649
PS-IBSIR	0.521	0.632	3.62	0.291	0.400	0.767	0.729	0.661	0.642
SW-FBP	25.1	35.1	57.3	16.0	25.8	42.8	0.543	0.537	0.472
SW-SIR	17.1	22.4	22.7	3.90	9.77	8.32	0.609	0.610	0.518
SW-IBSIR	0.632	0.637	3.96	0.272	0.404	0.889	0.731	0.679	0.628
EPS-IBSIR	0.372	0.564	3.74	0.239	0.356	0.830	0.730	0.700	0.632

PS stands for the phase-stepping simulation, SW for the sliding window simulation and SSEPS for the single-shot electromagnetic phase-stepping simulation. Reconstructions where performed using filtered backprojection (FBP), statistical iterative reconstruction (SIR) and interferogram-based SIR (IBSIR). The SIR and IBSIR approach performs better than the FBP method for all simulations, whereas superior behavior of IBSIR over SIR can only be claimed for the SW simulation. The SSEPS simulation that could only be reconstructed using IBSIR shows similar image quality as the IBSIR reconstruction of the SW scan.

The SW scan was also reconstructed using FBP, SIR, and IBSIR. Before the reconstruction using FBP and SIR, a sliding window interpolation between neighboring views had to be performed. The interpolated data was then processed the same way as the PS scan. The SW-FBP and SW-SIR reconstructions show severe artifacts, which could be expected as interpolation between neighboring views is only viable if the information content in one pixel changes slowly between neighboring views. These reconstructions are not shown, but image quality metrics can be found in Table 1. The SW-IBSIR reconstruction, see Fig. 1, does not require the intermediate interpolation step, but works directly on the interferograms and thus does not suffer from interpolation artifacts. The increase in angular sampling, compared to PS-IBSIR, results in reconstructions that do not show angular undersampling artifacts. On this note, very weak streak artifacts can be seen in the attenuation reconstruction that seem to be originating from the location of the high-frequency high-contrast objects in the scattering volume. In this instance, the algorithm was not able to attribute the changes in measured intensity to the correct signal.

The SSEPS simulation was only reconstructed using IBSIR as no other algorithm is able to reconstruct measurements acquired this way. The resulting SSEPS-IBSIR reconstructions show no image artifacts and provide a significantly improved image quality compared to the other results (see Table 1).

### Single-shot GBCT experiment

**Figure 2:**
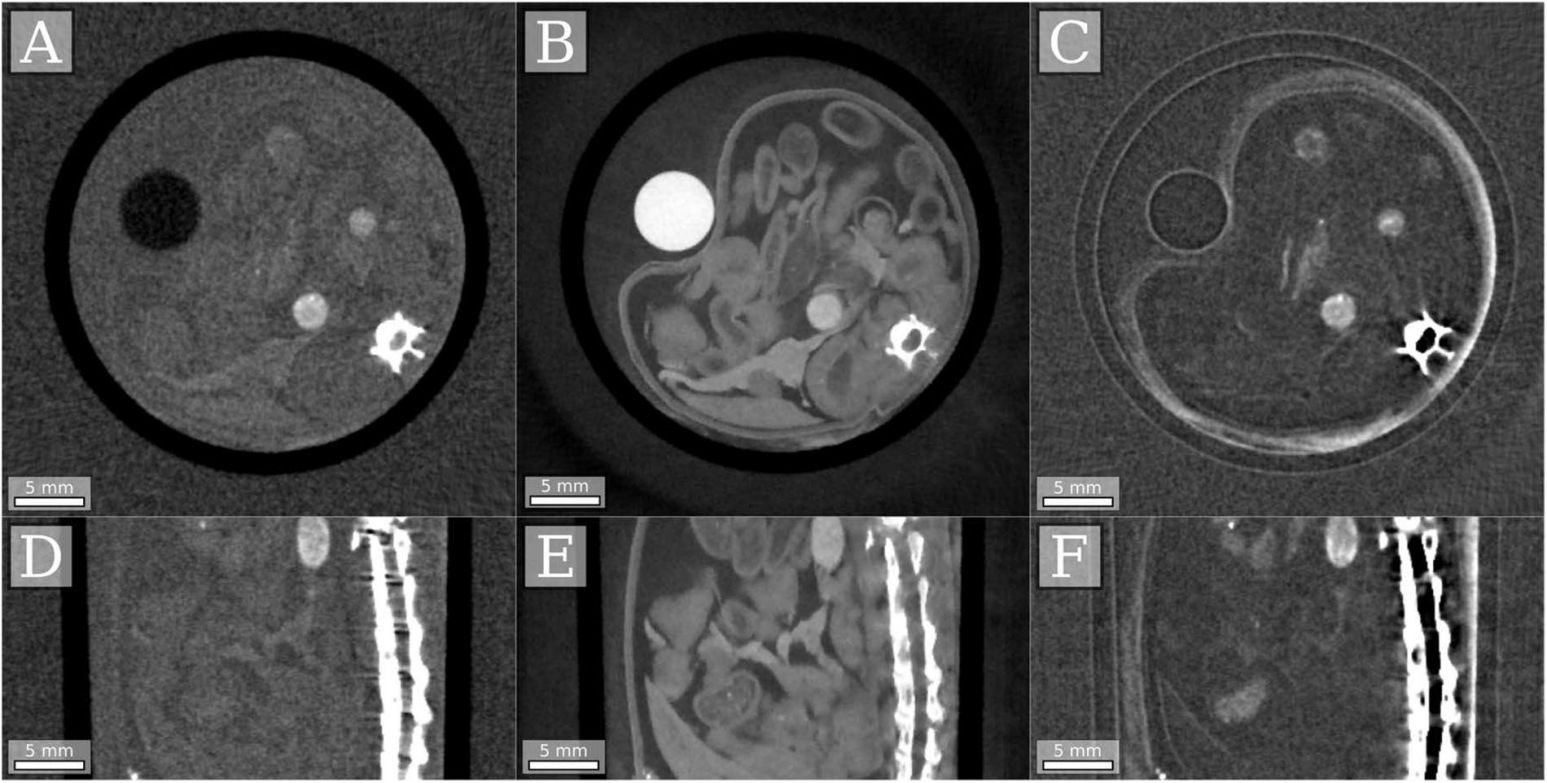
Axial (**A**–**C**) and sagittal (**D**–**F**) slices of the reconstructed attenuation coefficient (**A**,**D**), refractive index decrement (**B**,**E**) and linear diffusion coefficient (**C**,**F**) of *in-vitro* mouse intestines scanned using the described single-shot electromagnetic phase-stepping equivalent acquisition and the direct interferogram-based reconstruction algorithm.

As simulations can never reproduce all the effects present in a real measurement, experimental verification is necessary to fully illustrate the feasibility of single-shot GBCT without grating movement. Thus, an experiment was performed using a Talbot-Lau interferometer with a rotating anode X-ray tube radiation source. Instead of shifting the focal spot position by applying a magnetic field to the X-ray tubes electron beam, we physically shifted the sample by a known distance after each measurement. This is readily possible at most X-ray microscopy setups and (for small opening angles) produces virtually the same measurements as a focal spot shift. For the experiment the intestines area of a mouse fixed in formalin and placed in a falcon tube, was used as a sample, exhibiting a complex combination of attenuation, refraction and ultra-small-angle scattering. To avoid extreme refraction at the falcon tube-air interface the whole sample was placed in a water bath. For this scan 900 measurements were taken, equally distributed over 360 degrees, and the sample stage was cyclically stepped between 5 different positions, resulting in object shifts of 0, 4, 8, 2 and 6 pixels in the direction perpendicular to the rotational axis. The average period of the Moiré fringes in this direction was about 10 pixels. More details on the setup and scans can be found in the methods section. The result of the IBSIR reconstruction of this scan is shown in Fig. 2. The resulting reconstructions show no image artifacts and excellent image quality.

## Discussion

In this study we presented a strategy for data acquisition and reconstruction, specifically designed for GBCT, that is compliant with the demands of clinical applications, in particular a continuously rotating gantry. When employing the IBSIR algorithm on simulation and experimental data, the capability to reconstruct the three complementary signals directly from the raw interferograms was demonstrated. Further, we have illustrated that artifact-free reconstruction of GBCT using IBSIR is not only possible but can also be achieved with less than three measurements per pixel and per projection angle. To the best of our knowledge, no other reconstruction technique has been able to achieve this without a significant loss of resolution.

**Figure 3:**
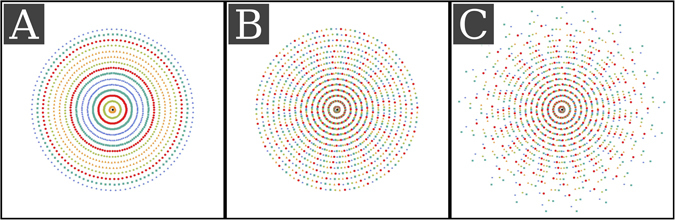
Visualization of the sampling of the proposed expanded Radon space for different acquisition patterns. The location of the symbols represents the location of the measurement in conventional Radon space using spherical coordinates. The color and shape represent the interferometer phase at which the measurement was taken. The visualizations where created for a setup with a 10° fan angle. (**A**) Shows a scan using a single fixed grating position. (**B**) Shows a sliding window phase-stepping scan using five grating positions. (**C**) Shows the pattern for the proposed single-shot electromagnetic phase-stepping (SSEPS) acquisition pattern.

To further analyze our results, one can consider the distribution of the measurements in Radon space^23^. Every single measured pixel of a conventional CT scan represents information at one point of the Radon transform of the measured object and the task of a CT reconstruction algorithm is to reconstruct the measured object by somehow calculating the inverse Radon transform using this information. In the case of GBCT, the Radon space representation of the measurements is not sufficient as each measured pixel is not only taken at a specific angular position and a specific distance from the center of the detector, but also at a specific phase position of the interferometer stepping curve. This expands the measurement space by an additional dimension in which sampling can be influenced by various effects: by shifting the gratings, by the presence and position of Moiré fringes or by the phase shift induced by the object itself. The task of GBCT reconstruction can now be understood as the inversion of this expanded radon space back to the object space (consisting of the 3D distributions of the linear attenuation coefficient, the refractive index, and the linear diffusion coefficient). All previous GBCT reconstruction algorithms solve this problem by first collapsing the phase dimension of the expanded Radon space into three separate conventional Radon spaces, then inverting them separately. For a conventional equidistant phase stepping scan this is easily done by the signal extraction step, as the whole extra dimension is sufficiently well sampled (at least three measurements equidistantly distributed along the extra dimension) for each point of the Radon space. Single shot methods can be understood as a collapse onto coarser grids or as interpolation of missing values on the expanded Radon space. For example, the Fourier analysis method^11, 12^, collapses onto a coarser grid along the detector pixel dimension of the Radon space and the sliding window interpolation technique^13^ uses nearest neighbor interpolation to fill missing values in the phase dimension with values taken from along the angular dimension. IBSIR on the other hand takes a very different approach because it directly inverts the expanded Radon space without first collapsing it. This can be highly beneficial for the image quality of the results, especially for single-shot acquisition protocols, because no interpolation during the signal extraction/collapsing is necessary, which necessarily reduces the possible spatial accuracy of the reconstructions. From the theory of compressed sensing it is known that the highest probability to correctly recover a signal in an underdetermined system can be achieved if the signal is sampled as nonuniform as possible. The distribution of the measurements along the conventional axes of the expanded Radon space is given by the setup geometry, but the distribution along the phase axis can be influenced by the acquisition protocol. Figure 3 illustrates a visualization of the extended Radon space for three different acquisition protocols simulated at a GBCT setup with a fan angle of 10 degrees and Moiré fringes with a period of five pixels. Figure 3A shows a simple scan with constant grating positions. This scan is very ill-suited for direct inversion as it is not well sampled along the phase dimension. Figure 3B,C depicts the expanded Radon space sampling for a SW scan and a SSEPS scan. From the color distribution it is evident that these scans are more diversely sampled along the phase dimension resulting in the high quality reconstruction results we observed in the reconstructions presented here. It is important to note, that this very specific analysis allows a plausible explanation of our results but is not a rigorous explanation. Nevertheless we think that during the design of future acquisition protocols for GBCT, the expanded Radon space sampling should be considered.

In summary, the proposed combination of the IBSIR algorithm with a SSEPS-like acquisition protocol successfully demonstrated that direct reconstruction algorithms strongly increase the general usability of GBCT imaging. The proposed acquisition protocol is perfectly suited for application with a clinical CT system equipped with a grating interferometer, because the required focal spot sweeping is already available in most modern clinical CT systems and the technique is well suited for a continuously rotating gantry. This is a milestone in a future transformation of grating-based phase-contrast and dark-field contrast X-ray computed tomography from an experimental tool to a robust and highly usable diagnostic imaging tool that may significantly improve clinical diagnostics.

## Methods

### Algorithms

For the signal extraction step, prior to FBP and SIR reconstructions, weighted least-squares fits of sine-curves to the measurements in each pixel were performed. The inverse of the fitting uncertainties of each fit was used as the statistical weight for the SIR.

The FBP reconstructions of the attenuation and dark-field projections were performed using a Ram-Lak filter with Hamming window and the FBP of the differential phase projections was reconstructed using a Hilbert filter^24^.

The regularizers that were used for both SIR and IBSIR penalized the difference between neighboring pixels:

8 $${R}_{\theta }=\sum _{j}\sum _{k\in {{\mathscr{N}}}_{j}}{w}_{jk}{\rm{\Psi }}({\theta }_{j}-{\theta }_{k},{\gamma }_{\theta }\mathrm{).}$$

The index *j* runs over all voxels and *k* runs over all voxels in the neighborhood ${{\mathscr{N}}}_{j}$ of *j*. *w*_*jk*_ is a distance dependent weight and as the potential function, Ψ(·), we used the Huber potential function^19^. This function increases quadratically for deviations from zero smaller than the Huber parameter *γ* and from that point on linearly for larger deviations. This results in a smooth solution that still preserves strong edges in the object. The regularization strength *β* heavily influences the noise level in each image and *γ* influences how steep an edge has to be to be preserved.The value of *γ* was set to the noise levels in the final reconstructions in all reconstructions. The regularization strengths *β* used during the simulations was set individually for each simulation such that the same moderate noise level per object property was achieved across all simulations. For the experimental data, *β* was set such that the image noise was significantly suppressed in each volume but no loss in resolution was visually observable.

For the attenuation and dark-field reconstructions using SIR the cost function was minimized using a momentum-accelerated OS-SPS algorithm^25^, whereas for the differential phase data the nonlinear conjugate gradient method was employed.

The IBSIR cost function was minimized using the L-BFGS algorithm^26^.

The line integrals in all reconstructions were evaluated using forward- and back-projectors based on projection matrices^27^. The differential line integrals were approximated as finite differences between neighboring line integrals.

### Numerical simulation

For the numerical simulations, we used the forward model presented in Eq. (3) to generate noise-free interferograms of a known phantom exhibiting attenuation, refraction and ultra-small-angle scattering. The reference phase of the interferograms was set to mimic vertical Moiré fringes with a frequency of 0.05 px^−1^ for the PS and the SW scan and a frequency of 0.38 px^−1^ for the SSEPS scan. A uniform visibility of 0.75 was used. This unrealistically high visibility was chosen to achieve similar CNR levels in the attenuation and dark-field image. The geometry constants *c*_*D*_ and *c*_*ϕ*_ were set to one. A 500 × 5 pixel noise free detector and parallel beam geometry were simulated. Afterwards Poisson noise corresponding to a total of 1 × 10^13^ emitted photons, evenly distributed on all measurements and pixels, was added to the projections. The phantom used for the simulations consists of three FORBILD head phantoms which are overlying and rotated by 120 degrees with respect to each other. The values in the phantom were rescaled to achieve a similar order of magnitude in the CNR across all images.

### Experimental setup

The experimental data was recorded using a symmetric Talbot-Lau interferometer^28^ with grating distances *G*0*G*1 = *G*1*G*2 = 85.7 cm. The three gratings G0, G1, and G2 are all made of gold on a silicon substrate with periods of 5.4 μm. The absorption gratings G0 and G2 have a height of 70 μm and 65 μm respectively. The phase grating G1 is designed to give a phase shift of at the system design energy of 27 keV. The source was an ENRAF Nonius FR 591 rotating anode X-ray tube with a Molybdenum target operated at 40 keV and 70 keV. The detector was a photon-counting PILATUS 100 k with 487 × 195 pixels. It uses an 1 mm thick silicon sensor with a quadratic pixel size of 172 × 172 μm^2^. The effective pixel size is 93 × 93 μm^2^. The source to detector distance was 2.56 m, resulting in a fan angle of 3.7°. The exposure time for every single interferogram was 6 s. The mean visibility of the interferometer was at 0.27 and the mean frequency of the Moiré fringes parallel to the rotational axis was 0.1 px^−1^.
